# Ferroptosis as an important driver of lupus

**DOI:** 10.1007/s13238-021-00892-1

**Published:** 2021-11-26

**Authors:** Chao Mao, Guang Lei, Li Zhuang, Boyi Gan

**Affiliations:** 1 Department of Experimental Radiation Oncology, The University of Texas MD Anderson Cancer Center, Houston, TX USA; 2 The University of Texas MD Anderson UTHealth Graduate School of Biomedical Sciences, Houston, TX USA

Systemic lupus erythematosus (SLE), the most common form of lupus, is a chronic autoimmune disorder characterized by a global loss of self-tolerance and hyper-activation of both innate and adaptive immune systems (Kaul et al., 2016). Neutrophils, the most abundant leukocytes in human blood, have a critical role in maintaining immune surveillance and tissue homeostasis, and its dysregulation is of high relevance to SLE (Ricklin et al., 2010). In patients with SLE, accelerated neutrophil death and the deficiency in clearing dying neutrophils cause nuclear and cytoplasmic antigen exposure, excessive production of type I interferon (IFN), and neutrophil extracellular trap (NET) release, subsequently inducing autoimmune responses (Garcia-Romo et al., 2011). Dysregulated neutrophil death is believed to be a major cause of SLE; however, the underlying mechanism of neutrophil death in SLE is not well-defined.

Ferroptosis is an iron-dependent form of regulated cell death induced by over-accumulation of lipid peroxides on cellular membranes (Dixon et al., 2012). Cells have evolved several ferroptosis defense mechanisms to counteract lipid peroxidation and fight against ferroptosis, prominent among which is glutathione peroxidase 4 (GPX4), which localizes in both cytosol and mitochondria and uses glutathione as its cofactor to convert toxic lipid hydroperoxides into corresponding non-toxic lipid alcohols (Yang et al., 2014; Mao et al., 2021). Ferroptosis is a double-edged sword in cellular life and has been associated with many diseases: while excessive ferroptotic cell death is highly relevant to acute kidney disease, cardiomyopathy, atherosclerosis, and neurodegenerative disorders, ferroptosis impairment can lead to tumor development (Jiang et al., 2021). Although our understanding of the pathological relevance of ferroptosis in human diseases has been significantly advanced in recent years, the potential link between autoimmune diseases and ferroptosis remained unknown. A recent study filled this knowledge gap by revealing that ferroptosis induced by *GPX4* transcriptional suppression represents a main form of neutrophil death in lupus (Li et al., 2021). This study further highlights the importance of targeting ferroptosis in SLE treatment in the future.

**Figure 1:**
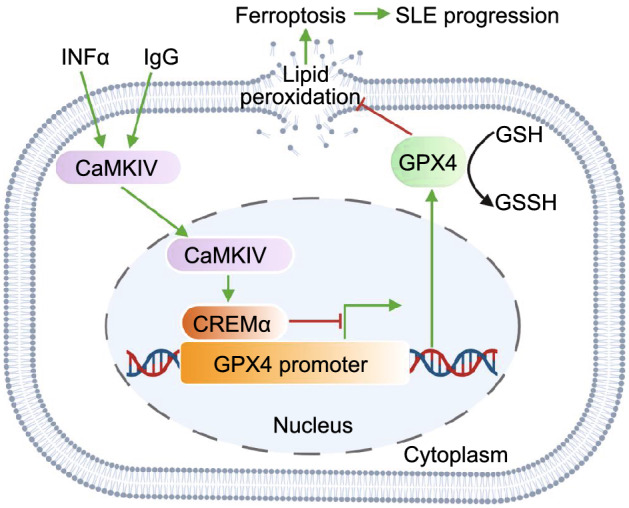
**Neutrophil ferroptosis promote SLE pathogenesis**. INFα and IgG in SLE serum activate the CaMKIV-CREMα axis, enhance the binding of CREMα to GPX4 promoter, suppress GPX4 expression, followed by increase lipid peroxidation levels. These lead to neutrophil ferroptosis and promote SLE pathogenesis. SLE: systemic lupus erythematosus; IFN-α: interferon-α; IgG: immunoglobulin G; CaMKIV: calcium/calmodulin kinase IV; CREMα: cAMP-responsive element modulator α; GPX4: glutathione peroxidase 4; GSH: glutathione; GSSH: oxidized glutathione

Through analyzing blood tests and viability detection from patients with autoimmune diseases, Li et al. (2021) confirmed that neutropenia (abnormally low levels of neutrophils) is a common feature in SLE; notably, SLE serum significantly promoted neutrophil death, further suggesting a key role of potential serum factors in regulating neutrophil death and neutropenia. Cytokine array analyses of inflammatory factors identified four cytokines (IFN-α, CXCL11, IL-12p40, and IL-23) with increased levels in SLE serum samples. Additional analyses revealed that blockade of IFN-α, but not other cytokines, restored the viability of neutrophils treated with SLE serum; conversely, the addition of IFN-α or autoantibodies from SLE serum compromised neutrophil viability, suggesting a role of IFN-α and immunoglobulin G (IgG) from SLE serum in mediating neutrophil death (Fig. 1).

NET release-induced neutrophil death (NETosis, another form of cell death) has been considered as a potential cause of SLE (Kenny et al., 2017). However, Li et al. (2021) showed that NETosis is not dominant in abnormal SLE neutrophil death; instead, this cell death exhibits features of ferroptosis, such as mitochondrial morphological changes and increased lipid peroxidation. Consistently, treatment with SLE serum, IFN-α, or IgG can significantly increase lipid peroxidation levels in neutrophils. Moreover, ferroptosis inhibitors liproxstatin-1 and deferoxamine significantly rescued SLE serum-induced neutrophil death; while other cell death inhibitors could also inhibit this cell death, their rescuing effects were not as statistically significant as those of ferroptosis inhibitors, indicating that ferroptosis inhibitors are the most effective cell death inhibitors in rescuing SLE serum-induced neutrophil death. To understand the underlying mechanisms, Li et al. (2021) performed RNA sequencing analyses and found that *GPX4* expression was decreased in neutrophils from SLE patients compared with those from health controls. Further investigations showed that *GPX4* expression was significantly reduced in neutrophils cultured with SLE serum, IgG, or IFN-α, suggesting that IFN-α in SLE serum or autoantibodies induce neutrophil ferroptosis potentially through downregulating *GPX4* expression.

So how is *GPX4* expression controlled in this context? cAMP-responsive element modulator α (CREMα) is a tissue-specific transcriptional repressor in immune cells. CREMα nuclear translocation and its binding ability on promoters are known to be governed by calcium/calmodulin kinase IV (CaMKIV). In SLE, increased nuclear translocation of CaMKIV has been shown to promote CREMα phosphorylation, contributing to aberrant T cell function (Juang et al., 2005). Through promoter sequence analyses, Li et al. (2021) identified a conserved CREMα binding site in the *GPX4* promoter, suggesting a potential role of CREMα in regulating *GPX4* expression. Consistently, increased nuclear accumulation and enhanced binding of CREMα on the *GPX4* promoter were observed in SLE neutrophils or healthy neutrophils cultured with SLE serum, IgG or IFN-α, compared with control neutrophils; further, genetic knockdown of *CREMα* reversed GPX4 expression suppression by IgG or IFN-α, while CREMα overexpression reduced *GPX4* expression. Together, these data indicate that SLE IgG and IFN-α inhibit *GPX4* expression through the CaMKIV-CREMα axis.

The authors also examined the relevance of neutrophil ferroptosis to lupus pathophysiology. Li et al. (2021) showed that lupus-prone mice exhibited reduced neutrophil viability and increased lipid peroxidation levels; moreover, compared with the NETosis inhibitor, ferroptosis inhibitors alleviated the disease progression more effectively in lupus-prone mice. To validate the role of GPX4 in neutrophil ferroptosis *in vivo*, Li et al. (2021) generated myeloid cell-specific *Gpx4* haploid-deficient (*Gpx4*^fl/wt^) mice. Similar to neutrophils in SLE patients, neutrophils from *Gpx4*^fl/wt^ mice showed impaired viability with excessive lipid peroxidation levels, and consequently, *Gpx4*^fl/wt^ mice developed lupus-like disease. Conversely, neutrophils from *Camk4* knockout (*Camk4*^−/−^) mice exhibited improved cell viability with lower lipid peroxidation levels, which correlated with elevated *Gpx4* levels in *Camk4*^−/−^ mice. Further, combined treatment with pristane and IFN-α adenovirus induced lupus-like disease in wild-type mice, but not in *Camk4*^−/−^ mice. Collectively, these results suggest that ferroptosis is an important cause of lupus, at least in mouse models.

Altogether, Li et al. (2021) reveal that serum autoantibodies and IFN-α repress *GPX4* expression and induce neutrophil ferroptosis through activating the CaMKIV-CREMα axis (Fig. 1). This study likely represents a conceptual breakthrough in the field of SLE research, not only enhancing our mechanistic understanding of lupus pathophysiology, but also indicating ferroptosis inhibitors as potential new therapeutic agents for SLE treatment. Of note, the involvement of IFNs in ferroptosis regulation has been reported previously. For example, cancer immunotherapy has been shown to enhance the effector function of CD8^+^ T cells, increase IFNγ release to downregulate SLC3A2 and SLC7A11 expression, resulting in reduced cystine uptake and increased ferroptosis in tumor cells (Wang et al., 2019). Furthermore, IFNγ and radiotherapy synergistically repress SLC7A11 expression, further enhancing lipid oxidation and ferroptosis in tumor cells (Lang et al., 2019). Several interesting questions also arise from this study. The underlying mechanisms by which autoantibodies and IFN-α promote CREMα nuclear translocation remains unknown. Further, infections are common in SLE and accounts for 25% to 50% of overall mortality (Petri, 1998). During infection process, host immune cells, such as neutrophils, release large amounts of reactive oxygen species (ROS) at infection sites (Winterbourn et al., 2016). Considering the intimate link between ROS and ferroptosis, the connection of infection and neutrophil ferroptosis remains an interesting topic to be investigated. Since ROS released by NADPH oxidase complex has been demonstrated to induce NETosis (Stoiber et al., 2015), whether there is any crosstalk between ferroptosis and NETosis remains another fascinating question in future studies. Finally, considering that SLE autoantibodies and IFN-α have relatively moderate inhibitory effects on *GPX4* expression, it is likely that other unknown mechanisms are involved in inducing neutrophil ferroptosis. Further studies are needed to address these important question.
